# Associations of SGLT-2i with Cardiorenal Outcomes Among Diabetics with Prostate Cancer on Hormone Therapy

**DOI:** 10.21203/rs.3.rs-4510870/v1

**Published:** 2024-07-26

**Authors:** Efstratios Koutroumpakis, Rushin Patel, Sumanth Khadke, Aram Bedrosian, Ashish Kumar, Yixin Kong, Brendan Connell, Jagriti Upadhyay, Sourbha S. Dani, Andrew W. Hahn, Christopher J. Logothetis, Sadeer Al-Kindi, Javed Butler, Anju Nohria, Sarju Ganatra, Anita Deswal

**Affiliations:** The University of Texas MD Anderson Cancer Center; Lahey Hospital and Medical Center; Lahey Hospital and Medical Center; Lahey Hospital and Medical Center; Cleveland Clinic Akron General; Lahey Hospital and Medical Center; Lahey Hospital and Medical Center; Lahey Hospital and Medical Center; Lahey Hospital and Medical Center; The University of Texas MD Anderson Cancer Center; The University of Texas MD Anderson Cancer Center; Houston Methodist Debakey Heart & Vascular Center; Baylor Scott & White Research Institute; Brigham and Women’s Hospital; Lahey Hospital and Medical Center; The University of Texas MD Anderson Cancer Center

**Keywords:** prostate cancer, hormone therapy, type II diabetes mellitus, SGLT2 inhibitors, cardiovascular outcomes

## Abstract

**Background:**

Studies have reported associations between prostate cancer, type II diabetes mellitus (T2DM) and cardiovascular disease in the context of treatment with hormone therapy (HT). This study aimed to assess the role of Sodium-Glucose Cotransporter-2 Inhibitors (SGLT2i) in preventing adverse cardiovascular and renal outcomes in diabetics with prostate cancer.

**Methods:**

Patients ≥ 18 years of age with T2DM and prostate cancer who received HT between August 1, 2013, and August 31, 2021, were identified using the TriNetX research network. Patients were divided into two cohorts based on treatment with SGLT2i or alternative antidiabetic therapies. The primary outcome was the composite of all-cause mortality, new onset heart failure (HF), acute myocardial infarction (MI), and peripheral artery disease over two years from HT initiation.

**Results:**

After propensity score matching, 2,155 patients remained in each cohort. The primary composite outcome occurred in 218 patients (16.1%) in the SGLT2i cohort versus 355 patients (26.3%) in the non-SGLT2i cohort (HR 0.689, 95% CI 0.582–0.816; p < 0.001). Furthermore, SGLT2i were associated with significantly lower odds of HF, HF exacerbation, peripheral artery disease, atrial fibrillation/flutter, cardiac arrest, need for renal replacement therapy, overall emergency room visits/hospitalizations and all-cause mortality.

**Conclusions:**

Use of SGLT2i for the treatment of T2DM among patients with prostate cancer on HT is associated with favorable cardiovascular, renal and all-cause mortality outcomes. This observation supports the hypothesis that a therapeutically relevant link exists between HT and cardiovascular disease in the context of prostate cancer.

## Introduction

Prostate cancer is the most common type of cancer among men, aside from skin cancer, with an estimated number of new cases in the US exceeding 288,000 in 2023 ^1^. Many patients with prostate cancer live beyond a decade from diagnosis and frequently die from non-prostate cancer related causes ^1,2^. Cardiovascular risk factors, including type 2 diabetes mellitus (T2DM) and cardiovascular disease are prevalent among patients with prostate cancer and represent a leading cause of mortality in this patient population ^2–4^. Hormone therapy (HT), which is the backbone of prostate cancer therapy, has also been associated with cardiotoxicity through alterations in body composition, lipid abnormalities and impaired glucose control ^5^. Even though the association of prostate cancer and HT with development of T2DM and cardiovascular disease is known for decades, a recent study reported that a significant portion of prostate cancer patients have undiagnosed or poorly controlled T2DM ^6,7^. This suggests that there is an unmet need for early diagnosis and appropriate treatment of T2DM tailored towards the prevention of cardiovascular disease.

Sodium-Glucose Cotransporter-2 Inhibitors (SGLT2i) have been associated with significant cardiovascular and renal benefits including prevention of heart failure (HF), renal failure and cardiovascular mortality, and have been recommended for the treatment of patients with T2DM at risk for or with established cardiovascular disease ^8^. Despite the significant potential benefits of SGLT2i in patients with prostate cancer, patients with active cancer were excluded from the clinical trials that established the cardiorenal benefits of SGLT2i ^9–12^. Therefore, their exact role and size of impact among patients with T2DM and prostate cancer treated with HT has not been established yet. We believe that improved understanding of the interaction of HT and SGLT2i in patients with prostate cancer will lead to a biologically sound strategy to mitigate the risk of cardiovascular disease. The aim of this study was to assess the incidence of adverse cardiovascular and renal outcomes in patients with prostate cancer on HT and T2DM treated with versus without SGLT2i using a large real-world database.

## Methods

### Study Oversight

Each of the authors contributed to various aspects of the study, including data analysis, manuscript development as well as review. The need for Institutional Review Board (IRB) approval was waived by Lahey Clinic IRB due to the use of deidentified data for the analysis. The study findings are reported per the Strengthening the Reporting of Observational Studies in Epidemiology (STROBE) guidelines for cohort studies.

### Data Source and Study Setting

This study utilized the TriNetX Analytics Network – Research Network, which is a collaborative health research network that draws upon de-identified electronic health records (EHRs) data from various participating healthcare organizations including academic medical centers, specialty physician practices, and community hospitals. The research network encompasses data from nearly 111 million patients. The data remains anonymized and is presented in aggregated form, however the network’s integrated analytics capabilities allow for the generation of patient-level data for tasks such as cohort selection and matching, as well as the analysis of the incidence and prevalence of events within a cohort. It also facilitates comparisons of characteristics and outcomes between matched cohorts. For further details about the database, additional information can be accessed online ^13^.

### Study Population and Design

The TriNetX research network was searched, and data curation was performed on August 31, 2023. A comparative retrospective cohort study was conducted, which included patients ≥ 18 years with pre-existing T2DM and a history of prostate cancer who received HT, which included GnRH analogs (leuprolide, triptorelin, goserelin, histrelin, relugolix, degarelix, abarelix) and/or androgen signaling inhibitors (enzalutamide, apalutamide, darolutamide, bicalutamide, flunamide, nilutamide and abiraterone) between August 1st, 2013 and August 31st, 2021. We elected to start our search from 2013 since earlier that year that the first SGLT2i was approved by FDA for the treatment of T2DM (canagliflozin, 3/29/2013) ^14,15^. We ended our search in 2021 to allow for two years of follow up. A two-year follow-up period was decided based on the follow-up period used in the previously published SGLT2i trials evaluating cardiovascular outcomes^11,12^. Patients included in this study were further categorized in two cohorts based on their use of SGLT2i (canagliflozin, dapagliflozin, empagliflozin). Various cardiovascular outcomes were obtained during the two years follow-up following the index event defined as initiation of HT for prostate cancer.

Patients with history of T2DM as well as prostate cancer were identified using two definitions based on the International Classification of Diseases, Tenth Revision (ICD-10) code. Identification of patients who were prescribed HT as well as SGLT2i was completed using the National Library of Medicine RxNorm terminology. Cohorts were matched using propensity score matching (PSM) using multiple baseline characteristics as deemed clinically significant. The Supplementary Appendix provides additional information on cohort definition criteria, analysis setup, outcome definitions and PSM.

### Study Endpoints/variables

#### Main Composite Outcome

The main composite outcome was all-cause mortality, HF, acute MI and peripheral artery disease (PAD) over two years from the index event of HT initiation. HF is any new diagnosis of HF and PAD any type of PAD as defined by the ICD codes provided in the supplementary appendix.

#### Secondary Outcomes

Secondary outcomes included individual outcomes of all-cause mortality, new onset HF, acute MI, PAD, HF exacerbation, LVEF < 50%, atrial fibrillation/flutter, cardiac arrest, ischemic stroke, need for renal replacement therapy and all-cause ER visits/hospitalizations. The outcomes were defined based on ICD or CPT codes and EHR extracted data. HF exacerbation was defined by the diagnostic codes plus need for IV diuretics.

#### Statistical Analysis

Patients with history of T2DM and prostate cancer who received HT were divided into 2 cohorts based on the use of SGLT2i: SGLT2i cohort and non-SGLT2i cohort. These two cohorts were compared using independent sample t-tests for continuous variables, reported as mean (range). Categorical variables are reported as counts (%) and compared using the Chi-square (χ2) test. To control for baseline differences in the patient cohorts, we performed 1:1 Propensity Score matching (PSM) for characteristics of clinical relevance utilizing a built-in algorithm that uses the greedy nearest-neighbor algorithm with a caliper of 0.1 pooled standard mean difference (SMD). Any characteristic with a SMD between the cohorts lower than 0.1 was considered well-matched. After propensity matching, time to event analysis reported as hazard ratios with 95% confidence intervals was performed for the primary outcome and odds ratios with 95% confidence intervals for the secondary outcomes using the χ2 for the measures of association. Absolute risk difference (ARD) was calculated as the subtraction of the absolute risk of the event in the treatment (SGLT2i) cohort and the absolute risk of the event in the control (non-SGLT2i) cohort. Furthermore, a sensitivity analysis was performed to evaluate the potential of significant confounding. For the sensitivity analysis we calculated E-values for the odds ratio as previously described ^16^. A large E-value means that significant unmeasured confounding would be needed to explain away an effect estimate while a small E-value means that little unmeasured confounding would be needed to explain away an effect estimate. Statistical analyses were completed using the TriNetX online platform using R for statistical computing.

#### Role of Funding Source:

No Funding Source is involved.

## Results

### Study Population

A total of 26,848 patients were identified with a history of T2DM and prostate cancer who received HT. Of the total patients, 2,741 patients received SGLT2i while 24,107 patients did not receive SGLT2i. After propensity score matching, 2,155 patients remained in each cohort (Table 1).

### Patient Demographics

Table 1 outlines the baseline characteristics of each cohort before and after propensity matching. The mean age (± SD) of patients in the SGLT2i cohort was 66.8 ± 12.8 and 57.8% of patients were White adults. Before propensity matching, patients treated with SGLT2i had a higher prevalence of comorbidities, including hypertension, hyperlipidemia, ischemic heart disease, cardiomyopathy, HF, atrial fibrillation/flutter, chronic lower respiratory diseases, and chronic kidney disease. The proportion of metformin, glucagon-like peptide-1 (GLP-1) agonists, as well as insulin use was higher in the SGLT2i cohort. All baseline characteristics between the two cohorts, including healthcare utilization, were propensity matched, with no residual difference (standard mean difference for all included covariates was < 0·1; Table 1).

### Main Composite Outcome:

**Figure 1:**
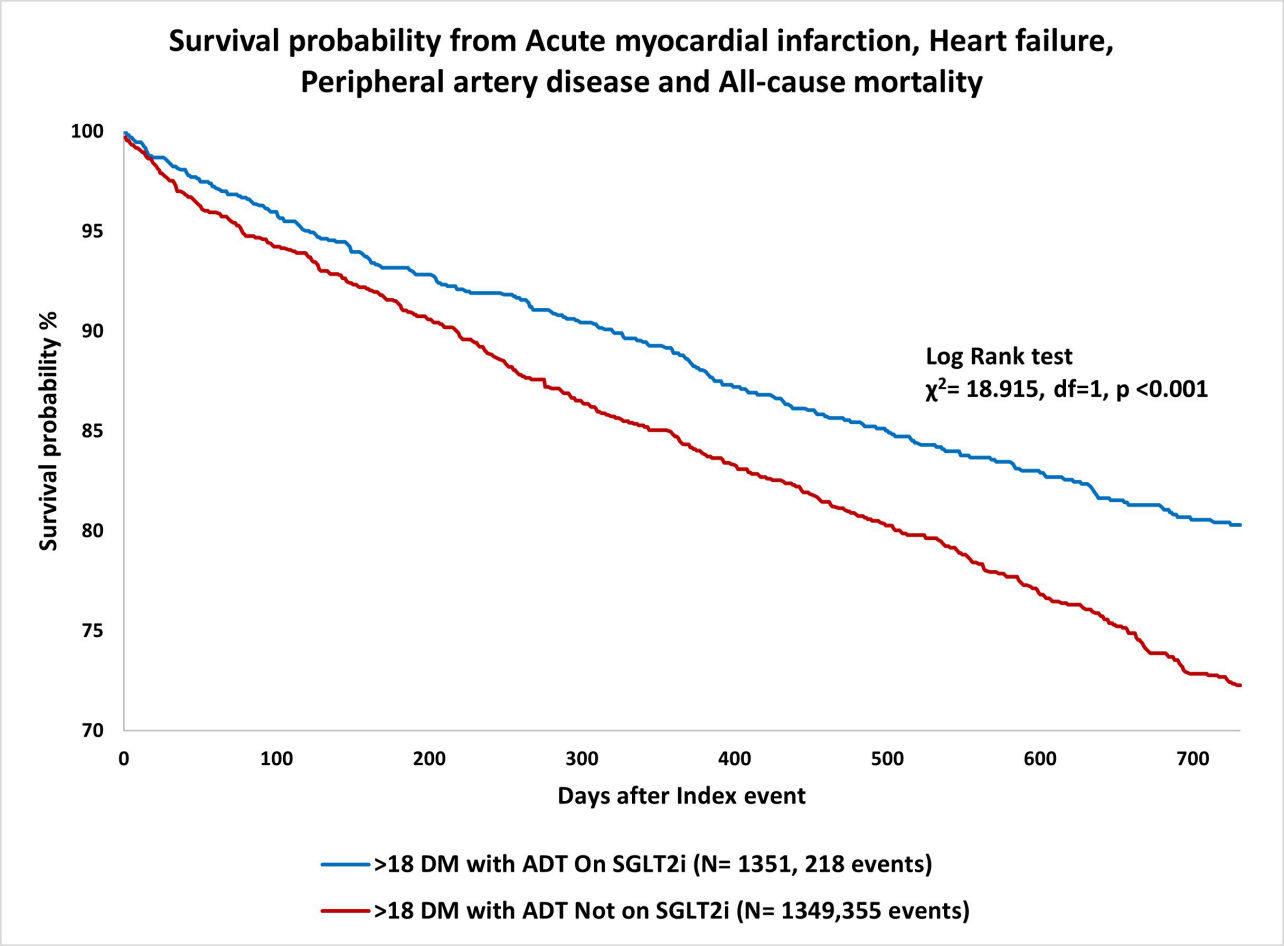
Kaplan-Meier curve demonstrating the association of SGLT2 inhibitors with higher survival probability from acute myocardial infarction, heart failure, peripheral artery disease and all-cause mortality among patients with prostate cancer on hormone therapy compared to propensity matched patients treated with other antidiabetic therapies.

Among patients with a history of T2DM and prostate cancer who received HT, patients who received SGLT2i had a lower risk of developing the composite outcome of all-cause mortality, HF, acute MI and peripheral artery disease over two years since initiation of HT, compared to propensity-matched controls who did not receive SGLT2i (HR 0.689, 95% CI 0.582–0.816; p < 0.001; Table 2; Fig. 1). The composite outcome occurred in 218 patients (16.1%) in the SGLT2i cohort as compared to 355 patients (26.3%) in the non-SGLT2i cohort (OR 0·54 CI 0·45 – 0·65; p < 0·001).

The E value of the Odds ratio for the primary outcome was 2.47 and the E value for the lower confidence interval was 2.88, both of which support stronger association of SGLT2i with the observed differences in outcomes (Table 2).

### Secondary Outcomes:

**Figure 2:**
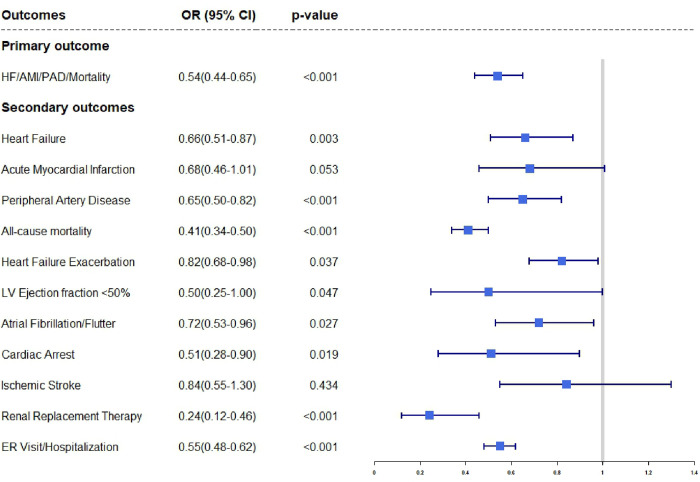
Forest plot demonstrating the odds ratios with 95% confidence intervals for primary and secondary outcomes in patients with prostate cancer on hormone therapy treated with SGLT2i for type II diabetes mellitus versus alternative antidiabetic agents.

Patients who were on SGLT2i had lower odds of having new onset HF (OR = 0.66, CI 0.51–0.87, p = 0·003); HF exacerbation (OR = 0.82, CI 0.68–0·99, p = 0.037); PAD (OR = 0.64, CI 0.50–0.82, p < 0.001). The SGLT2i cohort also had lowers odds of all-cause mortality (OR = 0.41, CI 0.34–0·50, p < 0.001) as well as lower odds of cardiac arrest (OR = 0.51, CI 0.29–0.90, p = 0.019). There were also lower odds of Atrial Fibrillation/Flutter in the SGLT2i cohort (OR = 0.72, CI 0.54–0.96, p = 0.027). The need for renal replacement therapy (RRT) was significantly lower in the SGLT2i cohort compared to the non-SGLT2i cohort (OR = 0.24, CI 0.12–0.46, p < 0.001). The patients who received SGLT2i had lower odds of healthcare utilization in the form of ER visits/hospitalizations (OR = 0.54, CI 0.49–0.62, p < 0.001; Table 2; Fig. 2).

Sensitivity analysis with E-values suggests stronger association of SGLT2i on observed outcomes and a low likelihood that differences in the outcomes are due to unmeasured confounders (Table 2).

## Discussion

To our knowledge, this is the first, large, real-world study evaluating the incidence of adverse cardiovascular and renal outcomes among patients with prostate cancer on HT treated with SGLTi compared to other agents for T2DM. We found that among patients with prostate cancer, SGLT2i treatment was associated with a significantly lower risk of developing the composite outcome of all-cause mortality, new onset HF, acute MI and PAD over two years since initiation of HT. In the analysis of individual outcomes, SGLT2i were associated with lower odds of new onset HF, HF exacerbation, PAD, atrial fibrillation/flutter, cardiac arrest, need for RRT, overall ER visits/hospitalizations and all-cause mortality.

Patients with prostate cancer represent a unique patient population with a high burden of comorbid cardiovascular conditions, including T2DM, as well as cardiovascular disease, which is a leading cause of death ^2–4^. The high prevalence of cardiovascular disease among patients with prostate cancer has been attributed not only to the coexistence of shared risk factors but also the effects of HT. HT is the backbone of prostate cancer therapy and it is used for as many as 50% of patients with prostate cancer at some point in their disease course. In 2006, Keating et al. were one of the first to report an association between GnRH agonists and increased incidence of DM, coronary heart disease, MI, and sudden cardiac death ^6^. Since then, several studies and clinical trials have confirmed that GnRH agonists, abiraterone, androgen receptor antagonists, and less so GnRH antagonists, are associated with increased incidence of adverse cardiovascular events. The mechanism of HT related cardiotoxicity includes hypogonadism-mediated alterations in body composition, with increase in adiposity and decrease in lean mass, lipid abnormalities (increase in triglycerides and LDL cholesterol) and impaired glucose control with decreased insulin sensitivity and subsequently elevated fasting serum glucose ^5^. These metabolic derangements lead to an increase in circulating proinflammatory adipokines and prothrombotic markers with subsequent vascular endothelial dysfunction, vascular inflammation, and adverse cardiovascular and renal events ^5^.

Over the last decade, several large clinical trials have shown that treatment with SGLT2 inhibitors is associated with favorable cardiovascular and renal outcomes in patients with T2DM at risk of or with established cardiovascular disease but also in patients with HF and chronic kidney disease (CKD) without T2DM ^9–12^. Empagliflozin was the first SGLT2i that received FDA approval in December of 2016 for reduction of cardiovascular death in adults with T2DM ^17^. Subsequently, in May of 2020 FDA approved dapagliflozin for reducing the risk of cardiovascular death and HF hospitalizations in patients with HF with reduced ejection fraction regardless of diabetes status, ^18^ and in April of 2021 dapagliflozin was approved to reduce the risk of kidney function decline in adults with CKD ^19^. More recently, in February of 2022, FDA approved empagliflozin to reduce the risk of cardiovascular death and hospitalizations in adults with HF regardless of ejection fraction ^20^. In addition to the above benefits, recent evidence suggests that SGLT2i may also reduce the incidence of atrial fibrillation/flutter ^21^. Our study findings further emphasize the importance of using SGLT2i in patients with T2DM to prevent adverse cardiorenal outcomes.

Considering that cardiovascular disease is a major cause of death among a large portion of men with prostate cancer, treatment with SGLT2i might have a great impact in improving mortality and morbidity. Furthermore, SGLT2i might be able to mitigate the risk of cardiotoxicity mediated by HT. Despite the significant potential benefits, patients with prostate cancer were excluded from the above-mentioned clinical trials and the exact role and size of impact of SGLT2i in preventing adverse cardiovascular and renal outcomes among patients with T2DM and prostate cancer treated with HT has not been examined. Our study is the first one to report a significant reduction in the risk of adverse cardiorenal outcomes among patients with prostate cancer on HT and T2DM treated with vs without SGLT2i. We hypothesize that by inducing glucosuria and natriuresis, SGLT2i improve hyperglycemia and hypertension, the two major risk factors contributing to the development of cardiovascular disease among patients with prostate cancer treated with HT ^22,23^. Furthermore, similar to patients with HF, it is likely that SGLT2i alter adipokine signaling and reduce inflammation, which could prevent HT-related cardiotoxicity ^22,23^. Based upon clinical observations and convergent data, our group has also postulated that a subset of prostate cancers is part of an “overlap syndrome” of age-related illnesses, including cardiovascular disease, with shared biology ^24^. The findings of this study suggest that SGLT2i warrant further investigation in this subset of patients since it has the potential to improve survival.

In addition to the favorable cardiorenal benefits of SGLT2i among patients with prostate cancer, preclinical studies have suggested functional expression of SGLT receptors in prostate adenocarcinomas and that treatment with SGLT2i might lead to favorable oncologic outcomes as well ^25^. Studies evaluating the role of SGLT2i in prostate cancer-specific outcomes in human subjects are currently underway (NCT04887935). Such trials may also be able to assess the cardiovascular benefits of SGLT2i in patients with prostate cancer irrespective of the presence of T2DM.

### Study Limitations:

Our study has several limitations including those inherent to observational studies such as selection bias. Most importantly, despite our efforts to carefully control for baseline differences in the SGLT2i versus non-SGLT2i cohort using propensity matching, unmeasured confounding may still exist. To eliminate this possibility, we performed a sensitivity analysis of patients with prostate cancer treated with vs without SGLT2i, the results of which indicated that the findings are unlikely to be explained by unmeasured confounders. Patients on SGLT2i may have also have socioeconomic differences related to access to medications, which cannot be assessed in this study. Differences in the type of HT that the two patient groups received, and the stage of their prostate cancer could not be assessed either, due to limitations of the database used. Furthermore, retrospective data curated from electronic medical records may be inaccurate or carry the risk of biases. This risk is somewhat mitigated by the large number of patients included in our study and the large effect size. Finally, this study did not assess side effects related to SGLT2i or prostate cancer specific outcomes.

## Conclusions

This study demonstrated that the use of SGLT2i for the treatment of T2DM was associated with significantly lower risk for developing the composite outcome of all-cause mortality, new onset HF, acute MI and PAD among patients with prostate cancer treated with HT. Clinical trials assessing the impact of SGLT2i in patients without T2DM or HF in reducing CV events associated with HT or prostate cancer outcomes are needed.

## Figures and Tables

**Table 1.** Baseline demographics and clinical characteristics of patients with prostate cancer on androgen deprivation therapy separated by treatment with or without SGLT2 inhibitors for type II diabetes mellitus, before and after propensity score matching.

	Before Propensity matching			After Propensity Matching		
Baseline Characteristics	SGLT2 inhibitor (n=2,741)	No SGLT2 inhibitor (n=24,107)	SMD	SGLT2 inhibitor (n=2,155)	No SGLT2 inhibitor (n=2,155)	SMD
**Demographics**						
Age, years	66.6 +/− 12.7 (Mean ± SD)	67.8 +/− 14.7 (Mean ± SD)	0.085	66.8 +/− 12.8 (Mean ± SD)	66.8 +/− 15.0 (Mean ± SD)	0.001
White	1,564 (57.1%)	14,552 (60.4%)	0.067	1,245 (57.8%)	1,259 (58.4%)	0.013
Non-Hispanic or Latino	1,892 (69.0%)	16,619 (68.9%)	0.002	1,475 (68.4%)	1,461 (67.8%)	0.014
**Comorbidities**						
Hypertension	2,211 (80.7%)	14,735 (61.1%)	0.440	1,680 (78.0%)	1,658 (76.9%)	0.024
Hyperlipidemia	2,055 (75.0%)	11,856 (49.2%)	0.551	1,527 (70.9%)	1,509 (70.0%)	0.018
Ischemic heart disease	1,066 (38.9%)	6,008 (24.9%)	0.303	788 (36.6%)	774 (35.9%)	0.014
Cardiomyopathy	304 (11.1%)	1,002 (4.2%)	0.264	206 (9.6%)	199 (9.2%)	0.011
Heart Failure	602 (22.0%)	2,690 (11.2%)	0.294	423 (19.6%)	414 (19.2%)	0.011
Ischemic stroke	402 (14.7%)	2,784 (11.5%)	0.092	311 (14.4%)	324 (15.0%)	0.017
Atrial fibrillation and flutter	456 (16.6%)	2,709 (11.2%)	0.156	325 (15.1%)	326 (15.1%)	0.001
Chronic lower respiratory diseases	745 (27.2%)	4,370 (18.1%)	0.218	538 (25.0%)	535 (24.8%)	0.003
Chronic kidney disease (CKD)	706 (25.8%)	4,094 (17.0%)	0.215	509 (23.6%)	477 (22.1%)	0.035
Metastatic cancer	743 (27.1%)	5,493 (22.8%)	0.100	606 (28.1%)	623 (28.9%)	0.017
**Procedures**						
Surgical Procedures on the Male Genital System	959 (35.0%)	5,984 (24.8%)	0.223	685(31.8%)	631(29.3%)	0.054
Radiation Oncology Treatment	744 (27.1%)	1,945 (8.1%)	0.517	469(21.8%)	443(20.6%)	0.030
**Medications**						
Statins	2,182 (79.6%)	11,330 (47.0%)	0.719	1,629 (75.6%)	1,640 (76.1%)	0.012
Antiarrhythmics	1,764 (64.4%)	10,092 (41.9%)	0.463	1,296 (60.1%)	1,287 (59.7%)	0.009
ACE inhibitors	1,435 (52.4%)	7,767 (32.2%)	0.416	1,077 (50.0%)	1,062 (49.3%)	0.014
Angiotensin II Inhibitors	1,069 (39.0%)	4,189 (17.4%)	0.494	754 (35.0%)	747 (34.7%)	0.007
Loop diuretics	1,572 (57.4%)	8,782 (36.4%)	0.429	1,175 (54.5%)	1,161 (53.9%)	0.013
Beta Blockers	1,671 (61.0%)	9,443 (39.2%)	0.447	1,247 (57.9%)	1,256 (58.3%)	0.008
Calcium Channel Blockers	1,236 (45.1%)	6,737 (27.9%)	0.362	908 (42.1%)	888 (41.2%)	0.019
Insulin	1,513 (55.2%)	6,742 (28.0%)	0.575	1,095 (50.8%)	1,127 (52.3%)	0.030
Metformin	1,971 (71.9%)	7,100 (29.5%)	0.938	1,439 (66.8%)	1,483 (68.8%)	0.044
Sitagliptin	628 (2.9%)	1,306 (5.4%)	0.518	391 (18.1%)	417 (19.4%)	0.031
Exenatide	114 (4.2%)	168 (0.7%)	0.226	68 (3.2%)	64 (3.0%)	0.011
Dulaglutide	314 (11.5%)	185 (0.8%)	0.458	126 (5.8%)	109 (5.1%)	0.035
Liraglutide	223 (8.1%)	258 (1.1%)	0.342	117 (5.4%)	112 (5.2%)	0.010
Semaglutide	119 (4.3%)	50 (0.2%)	0.280	40 (1.9%)	28 (1.3%)	0.045
Lixisenatide	18 (0.7%)	11 (0.0%)	0.103	10 (0.5%)	10 (0.5%)	<0.001
Glipizide	657 (24.0%)	2,103 (8.7%)	0.421	448 (20.8%)	467 (21.7%)	0.022
Aspirin	1,512 (55.2%)	8,525 (35.4%)	0.406	1,134 (52.6%)	1,139 (52.9%)	0.005
Apixaban	294 (10.7%)	750 (3.1%)	0.304	178 (8.3%)	170 (7.9%)	0.014
Warfarin	221 (8.1%)	2,010 (8.3%)	0.010	185 (8.6%)	202 (9.4%)	0.028
Rivaroxaban	162 (5.9%)	606 (2.5%)	0.170	104 (4.8%)	124(5.8%)	0.041
Clopidogrel	441 (16.1%)	2,191 (9.1%)	0.212	327 (15.2%)	348(16.1%)	0.027
Ticagrelor	87 (3.2%)	166 (0.7%)	0.181	45 (2.1%)	42(1.9%)	0.010
**Labs**						
Creatinine (mg/dL)	1.1 +/− 2.4	1.3 +/− 2.4	0.050	1.1 +/− 0.5	1.2 +/− 0.9	0.036
Hemoglobin (g/dL)	12.8 +/− 1.9	12.5 +/− 2.2	0.144	12.8 +/− 1.9	12.3 +/− 2.2	0.026
Cholesterol LDL ≥130 mg/dL	590 (21.5%)	3,317 (13.8%)	0.205	441(20.5%)	427 (19.8%)	0.016
BNP ≥150 pg/ml	257 (9.4%)	1,096 (4.5%)	0.191	174 (8.1%)	170 (7.9%)	0.007
NT-pro BNP ≥450 pg/ml	154 (5.6%)	728 (3.0%)	0.128	115 (5.3%)	109 (5.1%)	0.013
Hemoglobin A1c ≥7.0%	1,615 (58.9%)	5,329 (22.1%)	0.809	1,125 (52.2%)	1,111 (51.6%)	0.013
BMI ≥ 30kg/m^2^	972 (35.5%)	6,268 (26.0%)	0.206	734 (34.1%)	696 (32.3%)	0.037
Left Ventricular Ejection Fraction (LVEF) <40%	54 (2.0%)	130 (0.5%)	0.129	34 (1.6%)	33 (1.5%)	0.004

Abbreviations: SMD: Standardized Mean difference; SGLT2 – Sodium-glucose cotransporter 2;

**Table 2.** Comparison of primary and secondary outcomes of patients with prostate cancer on androgen deprivation therapy who were treated with or without SGLT2 inhibitors for type II diabetes mellitus.

Outcomes	SGLT2 inhibitor (n=2,155)	No SGLT2 inhibitor (n=2,155)	ARD (95% CI)	Odd Ratio (95% CI)	p-value	E-value for OR	E-value for lower bound CI of OR
**Composite of Outcomes (All-cause mortality/HF/AMI/PAD)**	218(16.1%) (n=1,351)	355(26.3%) (n=1,349)	−0.102 (−0.132,−0.071)	0.539 (0.446, 0.651) (HR 0.689, 95% CI 0.5820.816; p<0.001)	<0.001	2.47	2.88
**All-Cause Mortality**	168(7.8%) (n=2,155)	365(16.9%) (n=2,155)	−0.091 (−0.111,−0.072)	0.415 (0.342,0.503)	<0.001	4.31	5.33
**Heart Failure**	95(5.6%) (n=1,710)	141(8.1%) (n=1,734)	−0.026 (−0.043,−0.009)	0.665 (0.508,0.870)	0.003	2.4	3.33
**Acute Myocardial Infarction**	45(2.3%) (n=1,951)	65(3.3%) (n=1,952)	−0.010 (−0.021,0.000)	0.685 (0.466,1.008)	0.053	2.3	3.77
**Peripheral Artery Disease**	115(7.0%) (n=1,636)	171(10.5%) (n=1,633)	−0.034 (−0.054,−0.015)	0.646 (0.505,0.828)	<0.001	2.5	3.41
**Heart Failure Exacerbation**	243(11.3%) (n=2,155)	288(13.4%) (n=2,155)	−0.021 (−0.040,−0.001)	0.824 (0.687,0.989)	0.037	1.74	2.3
**Left Ventricular Ejection Fraction <50%**	12(0.57%) (n=2,083)	24(1.1%) (n=2,096)	−0.006 (−0.011,−0.000)	0.503 (0.252,1.003)	0.047	3.41	7.46
**Atrial Fibrillation/Flutter**	83(4.6%) (n=1,819)	113(6.2%) (n=1,816)	−0.017 (−0.031,−0.002)	0.721 (0.539,0.964)	0.027	2,12	3.18
**Cardiac Arrest**	18(0.83) (n=2,155)	35(1.6%) (n=2,155)	−0.008 (−0.014,−0.001)	0.510 (0.288,0.904)	0.019	3.33	6.6
**Ischemic Stroke**	40(1.98) (n=2,014)	47(2.3) (n=2,004)	−0.004 (−0.013,0.005)	0.844 (0.551,1.292)	0.434	1.67	3.04
**Renal Replacement Therapy**	11(0.5%) (n=2,155)	45(2.1%) (n=2,155)	−0.016 (−0.023,−0.009)	0.241 (0.124,0.466)	<0.001	7.8	16.15
**ER Visit/Hospitalization**	894(41.5%) (n=2,155)	1,216(56.4%) (n=2,155)	−0.149 (−0.179,−0.120)	0.547 (0.485,0.618)	<0.001	3.11	3.59

**Abbreviations:** SGLT2 – Sodium–glucose transport protein 2; ARD – Absolute risk difference; HF – heart failure; AMI – acute myocardial infarction; PAD – peripheral artery disease; ER – emergency room

## Data Availability

The data are available upon request by email to the corresponding authors.
